# Aerobic exercise improves clinical symptoms in people with Parkinson’s disease and its potential mechanism

**DOI:** 10.3389/fneur.2025.1658162

**Published:** 2025-10-02

**Authors:** Quanqing Wei, Hui Wang, Yuning Liu, Fengli Wang, Xin Wu, Chuanying Xu, Guiyun Cui, Jie Xiang

**Affiliations:** ^1^College of Medical Technology, Xuzhou Medical University, Xuzhou, China; ^2^The Second Clinical Medical College, Xuzhou Medical University, Xuzhou, China; ^3^The First Clinical Medical College, Xuzhou Medical University, Xuzhou, China; ^4^Department of Rehabilitation Medicine, Affiliated Hospital of Xuzhou Medical University, Xuzhou, China; ^5^Department of Neurology, Affiliated Hospital of Xuzhou Medical University, Xuzhou, China

**Keywords:** Parkinson’s disease, aerobic exercise, gut microbiota, bile acid, cytokines

## Abstract

**Background:**

An increasing body of research has identified a correlation between dysbiosis of the intestinal microbiota and the onset and progression of Parkinson’s disease. Furthermore, aerobic exercise has been shown to positively influence the regulation of intestinal microbiota. This study aims to examine the effects of aerobic exercise on the clinical symptoms of people with Parkinson’s disease and the underlying mechanisms.

**Methods:**

Twenty-six participants engaged in 8 weeks moderate-intensity aerobic exercise. The outcomes include the World Movement Disorder Society Unified Parkinson’s Disease Comprehensive Rating Scale part III and so on. Concurrently, fecal and blood samples were collected from the subjects.

**Results:**

After aerobic exercise, the MDS-UPDRS part III score decreased. There was a statistically significant increase in the relative abundance of *Clostridia* (*p* = 0.043) and *Roseburia* (*p* = 0.015). Diversity analysis revealed a statistically significant increase in both the Chao1 index and the Beta diversity index among people with Parkinson’s disease. Additionally, bile acid metabolic profiling indicated a significant reduction in fecal 7-ketolithocholic acid concentration in people with Parkinson’s disease. Serum levels of Taurochenodeoxycholic acid and Taurodeoxycholic acid were also decreased. T-cell factor assays demonstrated that the levels of the pro-inflammatory cytokines interleukin-1β and interleukin-8 decreased, while the level of the anti-inflammatory cytokine interleukin-4 increased.

**Conclusion:**

Aerobic exercise has been shown to enhance both motor and non-motor functions in people with Parkinson’s disease. The underlying mechanisms may involve the modulation of intestinal flora composition and diversity, bile acid metabolism, and inflammatory cytokine levels.

## Introduction

Parkinson’s disease is the second most common progressive neurodegenerative disorder in the world, which is characterized by bradykinesia, resting tremor, rigidity, and postural instability. Its non-motor symptoms include gastrointestinal dysfunction, sleep disorder, and so on. As the disease progresses, many patients experience a decline in their quality of life and may become unable to care for themselves, adversely affecting their physical, mental, and financial well-being, as well as that of their families. From 1990 to 2019, the burden of PD has tended to increase in most regions and countries worldwide (1). In addition, a national survey study of Chinese people aged >60 years showed that the prevalence of PD in this age group was 1.37% (2). Recent studies have found significant differences in the epidemiology of PD by age, gender, and region, with higher prevalence of PD in older adults and men (3). These data highlight the urgency of PD prevention and treatment. Currently, the clinical treatment of PD is still based on pharmacological treatment or deep brain electrical stimulation surgery, but long-term pharmacotherapy usually leads to adverse effects. In addition, non-pharmacological treatments include Tai Chi (4), Motor-Cognitive Dual-Task Training (5), aerobic exercise combined with transcranial direct current stimulation (6). Exercise therapy, especially, aerobic exercise, has received extensive attention as the complementary and adjuvant non-pharmacological therapy.

Aerobic exercise has been found to be neuroprotective in animal models of PD and to improve symptoms in people with Parkinson’s disease (7). Aerobic exercise, also known as endurance exercise, is a rhythmic and sustained movement of the body’s large muscle groups that increases heart rate and caloric demand with the aim of maintaining or improving physical health (8). Studies have shown a reduction in motor symptoms in people with Parkinson’s disease after aerobic exercise interventions (9). Similar results were observed in another study (10). In addition, aerobic exercise can also improve non-motor symptoms such as depressive symptoms and executive dysfunction in people with Parkinson’s disease (11). However, the mechanism by which aerobic exercise improves PD has not been fully clarified. It has been found that physical exercise promotes the production of neurotrophic factors, neurotransmitters, and hormones, and facilitates processes such as signaling, neuroplasticity, and autophagy (12). Wang et al. (13) found that 4 weeks of aerobic exercise can improve motor dysfunction in PD mice by modulating the striatal dopamine and glutamate Glu signaling pathways, attenuating the activity of extracellular signal-regulated kinase/mitogen-activated protein kinases signaling pathway in striatal medium spiny neurons. Johansson et al. (14) found that aerobic exercise improved cognitive control, increased functional connectivity in the anterior crustal nucleus and the right frontoparietal network, and reduced whole-brain atrophy. Several studies in humans (15–17) and animals (18–20) have shown that regular physical activity is associated with changes in gut microbiota. For example, studies by Fan et al. (19) and Wang et al. (20) both showed that aerobic exercise modulates gut microbes in PD mice. However, there are limited clinical trials in which aerobic exercise has been shown to improve PD symptoms by modulating the gut microbiota.

Gut microbiota and blood biochemistry tests may give us more insight into the mechanisms of aerobic exercise in Parkinson’s disease. Braak et al. proposed a hypothesis that Parkinson’s disease may originate in the gastrointestinal tract (21). This was validated by many subsequent studies (22). Recently, it has been recognized that gut microbial dysbiosis is highly associated with the pathogenesis of Parkinson’s disease and may be involved in the onset and development of PD through the “microbiota-gut-brain” axis (22, 23). Gut microbial dysbiosis is associated with abnormal immune responses, which are often accompanied by abnormal production of inflammatory cytokines (24). Dysbiosis of intestinal flora leads to elevated levels of lipopolysaccharide (LPS) in the gut, which in turn leads to intestinal inflammation, and LPS and inflammatory cytokines enter the bloodstream through the intestinal barrier, which in turn affects the peripheral immune system and microglia in the brain and promotes central nervous system pathology in PD (25). It was found that the gut microbiota and inflammatory cytokines were altered in people with Parkinson’s disease compared to healthy control populations. The abundance of different genera in the intestinal flora of people with Parkinson’s disease in *Firmicutes*, *Bacteroidetes*, *Actinobacteria*, and *Proteobacteria* was significantly different, and the abundance of *Bacteroidetes* and *Verrucomicrobia* was increased in the intestinal tracts of people with Parkinson’s disease, as compared to age- and sex-matched healthy control populations (26). In the peripheral blood of people with Parkinson’s disease, interleukin-1β (IL-1β), interleukin-2 (IL-2), interleukin-6 (IL-6), interleukin-10 (IL-10), tumor necrosis factor-*α* (TNF-α), and other inflammatory cytokines are elevated in concentration (27). There is a positive correlation between *Bacteroidetes* and plasma TNF-α concentration, and a positive correlation between *Verrucomicrobia* and plasma interferon-*γ* (IFN-γ) concentration, and altered plasma cytokine profiles associated with changes in the composition of the intestinal flora suggests that aberrant immune responses may contribute to the development of PD (28). In addition, intestinal flora is closely related to bile acids. Another important function of gut microbiota is to participate in the metabolism of bile acids, which include primary and secondary bile acids. Primary bile acids (for instance, cholic acid, chenodeoxycholic acid) are synthesized from cholesterol, and the intestinal flora can convert the primary bile acids into secondary bile acids (for instance, deoxycholic acid, lithocholic acid). The intestinal bacteria associated with secondary bile acid production include Eubacterium, Clostridium, Lactobacillus, and Bifidobacterium from the phylum *Firmicutes* (29). Li et al. (30) found that dysbiosis of gut microbiota may be associated with elevated secondary bile acids in Parkinson’s disease. The above evidence suggests that gut microbes may be a potential target for intervention to improve clinical symptoms of PD through aerobic exercise. However, there is limited research on the effects of aerobic exercise on the gut microbiota of people with Parkinson’s disease.

The above evidence suggests that gut microbes may be a potential target for intervention to improve clinical symptoms of PD through aerobic exercise. However, there is limited research on the effects of aerobic exercise on the gut microbiota of PD patients. Therefore, the main objective of this study was to investigate the effects of a short-term aerobic exercise intervention on clinical symptoms in PD patients and to explore the potential mechanisms through gut flora testing and blood biochemical tests (bile acid metabolic profiles and cytokines). We hypothesized that short-term aerobic exercise interventions could improve clinical symptoms in PD patients, and potential mechanisms may include modulation of the relative abundance and diversity of intestinal flora, modulation of bile acid metabolite levels, and modulation of T-cell factors in PD patients.

## Materials and methods

### Design

Twenty-six participants with Parkinson’s disease who attended the Department of Neurology or Parkinson’s Center at the Affiliated Hospital of Xuzhou Medical University were recruited. All participants who participated in this study were engaged in an 8-week moderate-intensity aerobic exercise training intervention for 30 to 50 min at least three times per week. The primary outcome was MDS-UPDRS III and the secondary outcomes included Berg Balance Scale (BBS), the Freezing Gait Questionnaire (FOG-Q), Non-Motor Symptoms Scale (NMSS), Mini-Mental State Examination (MMSE), Montreal Cognitive Assessment (MoCA), Hamilton Anxiety Scale (HAMA), Hamilton Depression Scale (HAMD), Cleveland Constipation Score, Pittsburgh Sleep Quality Index (PSQI), and Parkinson’s Disease Quality of Life Questionnaire (PDQ-39). Outcome measures were collected both at baseline and at 8 weeks.

### Participants

Inclusion criteria included patients with Parkinson’s disease diagnosed by a specialist in strict accordance with the clinical diagnostic criteria for PD published by the International Movement Disorder Society in 2015 or the 2016 edition of the Chinese diagnostic criteria for Parkinson’s disease; aged between 30 to 75 years old; with Hoehn and Yahr stage 1–3; without contraindications to exercise; and signed an informed consent form.

Exclusion criteria included patients with secondary Parkinson’s syndrome or Parkinson’s superimposed syndrome; patients with severe heart, liver, and kidney disease; patients with severe cognitive dysfunction, severe audiovisual dysfunction, severe anxiety and depression and other diseases that make them unfit to do aerobic exercise; patients with chronic gastrointestinal diseases or long-term regular use of antibiotics, microecological agents or other medications that may associated with an altered gut microbiota; patients with medication irregularity; and patients with exercise habits.

### Intervention

During this training study, all subjects were asked to exercise on a stationary home exercise bike at 40 to 60% of the individual heart rate reserve (HRR) at least 3 times per week, beginning with a 5-min warm-up, followed by 30 to 50 min of exercise, and ending with a cooldown of 5 min. The HRR was calculated based on individual maximal and resting heart rates assessed during the cardiorespiratory exercise test. Participants underwent cardiorespiratory exercise testing to assess their aerobic capacity and cardiorespiratory fitness levels and to establish target exercise volumes and target heart rates. Assessments were performed at baseline and after the 8-week intervention period. The formula for calculating the target heart rate is: Target HRR = [(max HR - resting HR) × (40% ~ 60%) + resting HR]. We chose this type of exercise because it is relatively safe and participants have a lower risk of falling while exercising at home without supervision.

Before the exercise, participants were taught how to use the exercise bike and exercise bracelet and to use the exercise bracelet to monitor their heart rate and adjust the intensity of the exercise based on their heart rate or the Borg scale (6–20 score). Participants recorded their exercise daily on an exercise diary sheet. Heart rates were uploaded to an app for the researcher to view. The researcher followed up twice a week to monitor and supervise the implementation of the intervention program.

## Outcome measures

### Clinical assessments

The primary outcome: The World Movement Disorders Society Unified Parkinson’s Disease Rating Scale (MDS-UPDRS). The scale consists of four sections, Part III is the primary outcome: Part I: Non-Motor Aspects of Experiences of Daily Living, to assess the impact of non-motor symptoms on the patient’s daily life. Part II: Motor Aspects of Experiences of Daily Living, to capture the patient’s perspective on how motor symptoms impact their ability to perform daily activities. This patient-reported section covers difficulties with tasks such as speech, salivation, chewing, eating, dressing, hygiene, handwriting, turning in bed, tremors, and walking. Part III: Motor Examination, it involves a systematic examination of a broad range of motor functions. Part IV: Motor Complications, to evaluate the presence and functional impact of motor complications that often arise from long-term levodopa therapy.

The secondary outcomes:

1. Berg Balance Scale (BBS), to objectively assess a person’s static and dynamic balance abilities, and to determine their risk of falling. It evaluates performance on 14 common tasks of varying difficulty.
2. the Freezing Gait Questionnaire (FOG-Q), to assess the severity and impact of freezing of gait (FOG), a common phenomenon in Parkinson’s disease where patients feel their feet are “glued to the floor” and have difficulty initiating or continuing walking.
3. Non-Motor Symptoms Scale (NMSS), to comprehensively identify and measure the severity of a wide range of non-motor symptoms (e.g., cardiovascular issues, sleep, mood, cognition, gastrointestinal problems) that are prevalent in Parkinson’s disease but are not related to movement.
4. Mini-Mental State Examination (MMSE) and Montreal Cognitive Assessment (MoCA), to screen for and evaluate cognitive impairment. The MMSE is a general screen for dementia. The MoCA is more sensitive for detecting mild cognitive impairment, particularly in domains like executive function and attention, which are often affected early in Parkinson’s disease.
5. Hamilton Anxiety Scale (HAMA) and Hamilton Depression Scale (HAMD), to rate the severity of anxiety (HAMA) and depressive (HAMD) symptoms in individuals with various medical and psychiatric conditions.
6. Cleveland Constipation Score, to assess the severity of chronic constipation by evaluating the frequency and difficulty of bowel movements, the need for manual assistance, and the feeling of incomplete evacuation.
7. Pittsburgh Sleep Quality Index (PSQI), to measure sleep quality and disturbances over a one-month time interval. It differentiates “poor” from “good” sleep by assessing seven components including latency, duration, efficiency, and daytime dysfunction.
8. Parkinson’s Disease Quality of Life Questionnaire (PDQ-39), to assess how often PD patients experience difficulties across 8 dimensions of daily living (e.g., mobility, activities of daily living, emotional well-being, stigma). It is a patient-reported outcome measure that provides a profile of the impact of the disease on health-related quality of life.

Assessments were performed at baseline and at 8 weeks.

### 16S rRNA amplicon sequencing

Fecal samples were collected at baseline and following 8 weeks of aerobic exercise. Participants were provided with 2 or 3 plastic devices to collect stool samples, which were stored at −80°C until DNA extraction. Participants collected their stool samples at the hospital or home, and they were asked to hand in the sample within one hour. Because most of the participants lived near the hospital, all samples were stored at −80°C in time. And transported to Novozymes for 16S rRNA amplicon sequencing using dry ice.

### Bile acid metabolism

Stool and serum samples were collected at baseline and 8 weeks post-intervention and all samples were stored in a − 80° refrigerator after collection, and then sent to the Key Laboratory of New Drug Research and Clinical Pharmacy of Jiangsu Province for testing, and a triple quadrupole liquid tandem mass spectrometry (AB SCIEX Triple Quad™ 5,500 LC/MS/MS system) was used to detect the bile acid content of the stool and serum samples of patients with PD.

### T-cell factor assay 12 (flow cytometry)

Blood samples collected in both baseline and post-test were centrifuged at 3000 × g for 10 min at 4°C immediately after collection. The plasma was then stored at −80°C for further assays. The plasma was then sent to the Department of Hematology for 12 T-cell factor tests: interleukin-1β (IL-1β), interleukin-2 (IL-2), interleukin-4 (IL-4), interleukin-5 (IL-5), interleukin-6 (IL-6), interleukin-8 (IL-8), interleukin-10 (IL-10), interleukin-12p70 (IL-12p70), interleukin-17 (IL-17), interferon-alpha (IFN-*α*), interferon-gamma (IFN-*γ*), and tumor necrosis factor-alpha (TNF-α).

## Statistics

The sample size was calculated based on the primary outcome of MDS-UPDRS-III. According to the previous literature (31), people with Parkinson’s disease demonstrated a mean reduction of 4.08 in their MDS-UPDRS-III scores following an 8-week cycling intervention, with a standard deviation of 5.68. With a two-sided *α* set at 0.05 and a power of 90%, the PASS 15 software computed a sample size of 23. Factoring in an estimated 10% attrition rate for study visits, the final number of participants required for inclusion was determined to be 26.

All statistical analyses were performed in IBM SPSS statistics (version 27), GraphPad. Prism.10.1.2 and R package (version 4.4.2). Data were checked for normality with the use of the Shapiro–Wilk test. For outcomes with normally distributed data, means and standard deviations were calculated for each variable. Differences between groups were compared using the paired samples t-test or paired Wilcoxon signed rank test. During data processing, outliers (i.e., values outside the mean ± 2 times the standard deviation) in the bile acid metabolic profiles and T-cell factor assays were excluded according to the 2σ criterion, except for possible factors such as instrumental or sample mishandling. Correlations between gut microbiota abundance, scale scores, bile acid, and cytokine level changes were examined using Spearman’s correlation. Differences were considered statistically significant at *α* = 0.05 and *p* < 0.05.

## Results

### Flow of participants through the study

**Figure 1:**
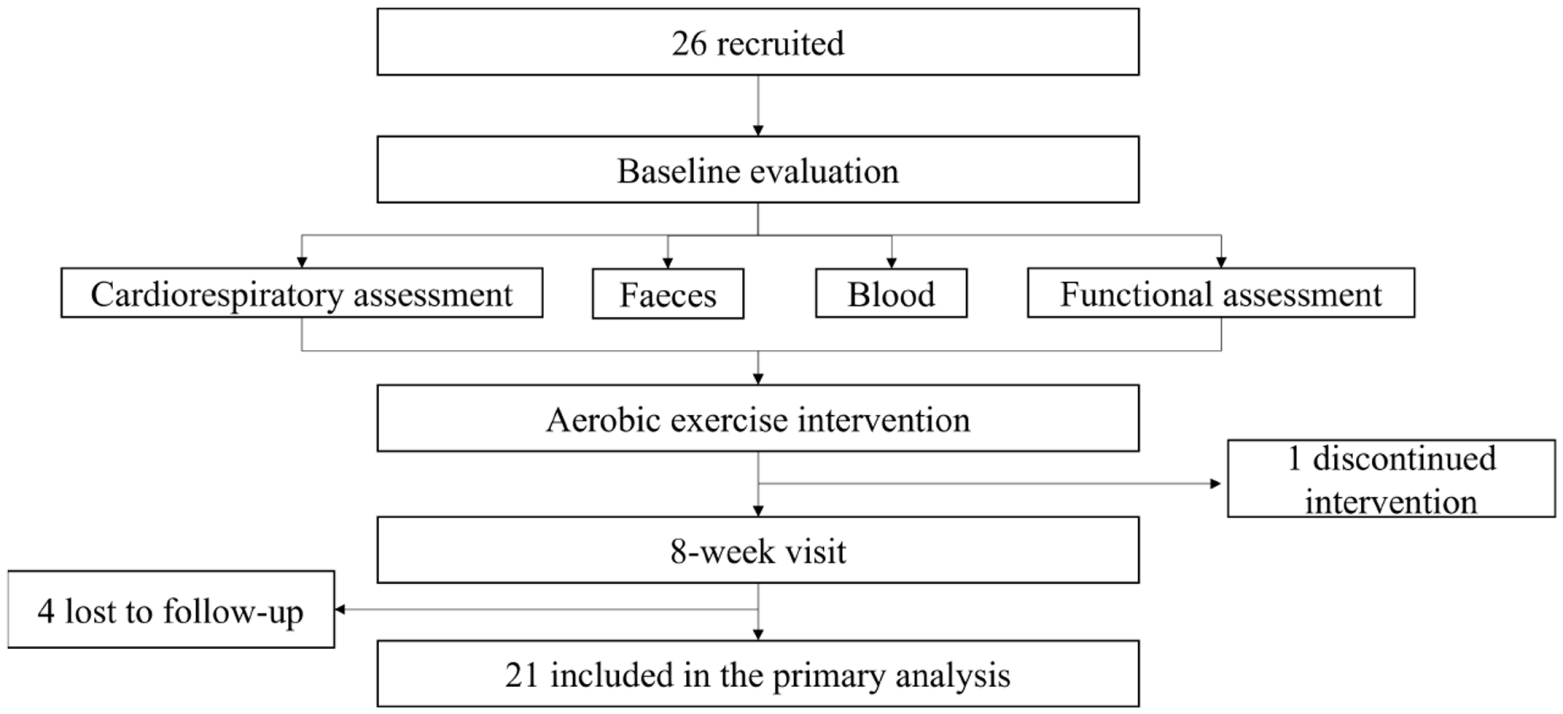
The flowchart of subject recruitment and follow-up.

Twenty-six (14 males, 12 females) were recruited. The flow participants and loss to follow-up through the research is shown in Figure 1. We recruited 26 (14 males, 12 females), of which 1 subject could not continue to complete the next exercise and follow-up visits due to low back pain, 4 subjects could not complete the follow-up visits due to their own reasons, 21 subjects completed the exercise intervention and all the assessments and had no adverse effects.

### Baseline characteristics of participants

The demographics of the subjects are presented in Table 1. The mean age was 61 years, the median disease duration was 5 years and the median years of education was 11 years. In addition, the median LEDD was 624 mg, all patients were instructed not to change their medication dosage or schedule throughout the study period. No significant adjustments in medication were reported during the trial.

**Table 1:** Baseline characteristics of participants.

Characteristic	Participants (*n* = 26)
Gender, *n* (%) Male Female	14 (54) 12 (46)
Age (y), mean (SD)	61 (9.5)
Height (cm), mean (SD)	164 (8.8)
Weight (kg), mean (SD)	68 (12)
BMI, mean (SD)	25 (3.5)
Disease duration (y), median (IQR)	5.0 (5.0)
H-Y, *n* (%)	
1	5.0 (19)
1.5	8.0 (31)
2	9.0 (35)
2.5	2.0 (7.7)
3	2.0 (7.7)
Education years (y), median (IQR)	11 (4.7)
LEDD (mg), mean (SD)	624 (309)

BMI, body mass index; H-Y, Hoehn-Yahr staging; LEDD, levodopa daily dose.

### The effect of aerobic exercise on clinical symptoms

**Figure 2:**
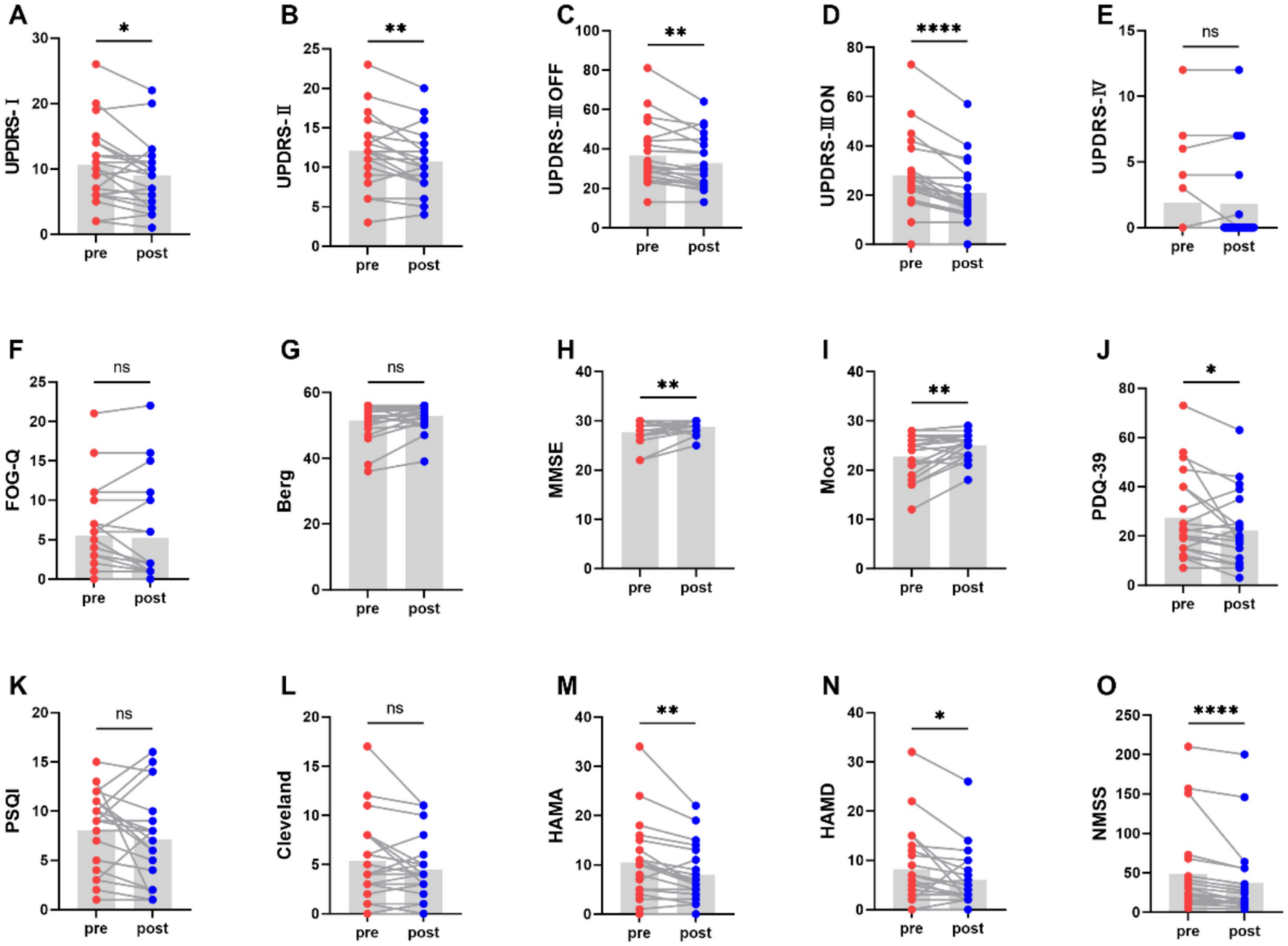
Comparison of scores on motor and non-motor symptom scales before and after aerobic exercise intervention, *P < 0.05; **P < 0.01; ***P < 0.001; ****P < 0.0001.

Figure 2 and Table 2 represents the comparison of scores on motor and non-motor symptom scales before and after aerobic exercise intervention. After aerobic exercise intervention, the scores of MDS-UPDRS part III OFF-state scores significantly decreased after the aerobic exercise intervention compared with those at baseline (*p* = 0.005). There are similar results for MDS-UPDRS part III ON-state scores (*P*<0.0001). And MDS-UPDRS part I and II were also significantly decreased (*p* = 0.021, *p* = 0.005). However, MDS-UPDRS part IV was not statistically significant (*p* = 1.000). Berg scores of PD patients tended to increase after the intervention but the difference was not statistically significant (*p* = 0.066). Meanwhile, there was a tendency for the Freezing Gait Questionnaire score to decrease after the intervention but the difference was also not statistically significant (*p* = 0.354). These findings suggest that aerobic exercise can improve the motor symptoms of people with Parkinson’s disease.

**Table 2:** Comparison of scores on motor and non-motor symptom scales before and after aerobic exercise intervention.

Scales	Pre	Post	
MDS-UPDRS1	10.67 ± 5.98	9.05 ± 5.30	0.021
MDS-UPDRS2	12.05 ± 4.50	10.67 ± 3.88	0.005
MDS-UPDRS3OFF	36.76 ± 15.97	32.48 ± 12.81	0.005
MDS-UPDRS3ON	28 ± 15.70	21.38 ± 13.52	0.000
MDS-UPDRS4	1.86 ± 3.40	1.81 ± 3.46	1.000
FOG-Q	5.52 ± 5.64	5.19 ± 6.37	0.354
Berg	51.38 ± 5.64	52.76 ± 4.07	0.066
Moca	22.67 ± 4.33	25.05 ± 2.73	0.002
PDQ-39	27.48 ± 17.32	22.24 ± 14.86	0.027
PSQI	8.05 ± 3.93	7.1 ± 4.7	0.236
Cleveland	5.33 ± 4.26	4.52 ± 3.23	0.179
HAMA	10.48 ± 7.93	8 ± 5.75	0.002
HAMD	8.24 ± 7.98	6.05 ± 5.98	0.035
NMSS	48.19 ± 56	37.24 ± 48.94	0.000

Moreover, the scores of MMSE and Moca increased (*P*<0.01), indicating that aerobic exercise can also enhance the cognitive function. And the scores of PDQ-39 was significantly decreased (*p* = 0.027). However, PSQI and Cleveland was not statistically significant (*p* = 0.236, *p* = 0.179). Meanwhile, aerobic exercise was also effective in alleviating the anxiety and depression of people with Parkinson’s disease, as demonstrated by decrease in the HAMA and HAMD score (*p* = 0.002, *p* = 0.035). Additionally, the NMSS score significantly decreased (*P* < 0.0001), further highlighting the beneficial effects of aerobic exercise on non - motor symptoms of people with Parkinson’s disease.

In addition, subgroup analyses were done in order to compare differences in clinical performance between PD patients of different genders at baseline and post-intervention, and the results suggest that there was no significant difference in the clinical presentation of PD males and females at baseline and post-intervention in this study (Table 3).

**Table 3:** Subgroup analysis on motor and non-motor symptom scales between male and female participants.

Scales	Pre			Post		
	Males	Females	*P*	Males	Females	*P*
MDS-UPDRS 1	9.58 ± 6.64	12.11 ± 4.96	0.351	8.08 ± 5.73	10.33 ± 4.66	0.348
MDS-UPDRS 2	12.33 ± 4.91	11.67 ± 4.15	0.746	11.08 ± 4.1	10.11 ± 3.72	0.583
MDS-UPDRS 3 OFF	38.83 ± 16.45	34 ± 15.83	0.507	36 ± 13.51	28.56 ± 13.13	0.221
MDS-UPDRS 3 ON	28.58 ± 17.89	27.22 ± 13.23	0.85	22 ± 14.19	19.78 ± 10.38	0.697
MDS-UPDRS 4	2.17 ± 3.83	1.44 ± 2.88	0.642	1.92 ± 3.87	1.67 ± 3.04	0.875
FOG-Q	4.58 ± 4.8	6.78 ± 6.69	0.391	4.58 ± 5.11	6 ± 8.02	0.626
Berg	52.42 ± 5.87	50 ± 5.34	0.344	52.75 ± 4.81	52.78 ± 3.11	0.988
MMSE	28.08 ± 2.35	27.11 ± 2.09	0.339	28.83 ± 1.11	28.67 ± 1.5	0.773
Moca	23.75 ± 3.44	21.44 ± 5.36	0.245	25.58 ± 2.27	24.33 ± 3.24	0.311
PDQ-39	22.58 ± 17.3	34 ± 15.95	0.139	21.92 ± 17.23	22.67 ± 11.99	0.912
PSQI	6.92 ± 3.96	9.56 ± 3.54	0.131	6.33 ± 4.46	8.11 ± 5.09	0.405
Cleveland	4.17 ± 3.71	6.89 ± 4.65	0.152	3.83 ± 3.38	5.44 ± 2.96	0.269
HAMA	7.92 ± 6.04	14 ± 9.03	0.079	6.5 ± 5	10 ± 6.36	0.174
HAMD	5 ± 4.41	12.56 ± 9.79	0.055	4 ± 3.3	8.78 ± 7.73	0.068
NMSS	36.33 ± 56.01	64 ± 55.09	0.273	30.42 ± 54.21	46.33 ± 42.24	0.475

### Aerobic exercise modulates gut microbiota composition in people with Parkinson’s disease

By sequencing the V4 region of the 16S rRNA gene of bacteria from 42 fecal samples from 21 subjects, 123 species of 12 phyla, 19 orders, 46 phyla, 79 families, 195 genera were identified pre-intervention and 130 species of 14 phyla, 21 orders, 50 phyla, 89 families, 199 genera were identified post-intervention.

**Figure 3:**
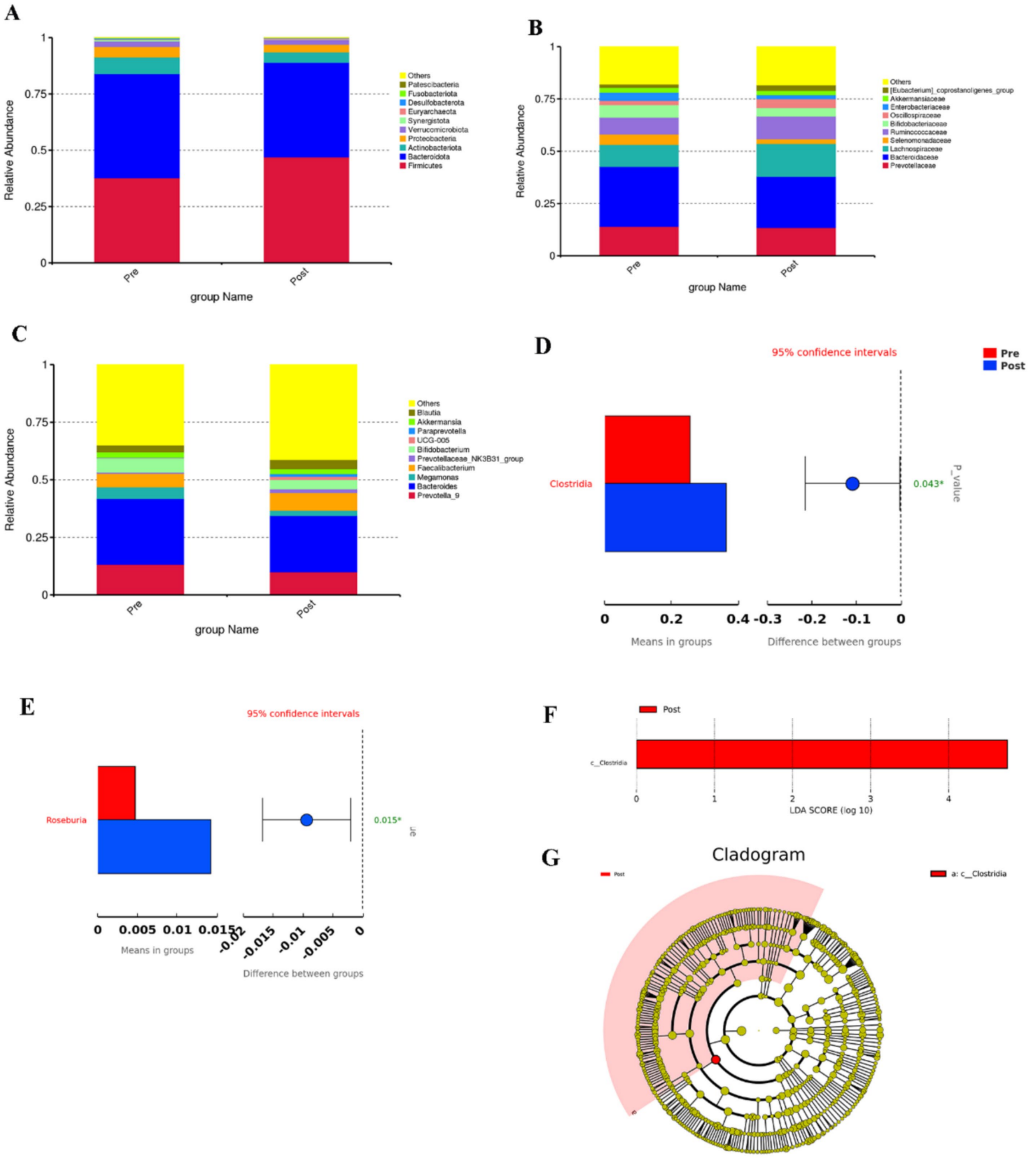
**(A–C)** Top 10 bar charts of relative abundance at the phylum, family and genus levels before and after the aerobic exercise intervention, **(D)** Plot of species differences between groups before and after aerobic exercise intervention at the order level, **(E)** Species differing between groups at the genus level, **(F)** Histogram of the distribution of LDA values, **(G)** Evolutionary branching in the Lefse analysis.

At the phylum level, the four most abundant bacterial phyla identified were constituted by *Firmicutes*, *Bacteroidetes*, *Actinobacteria*, and *Proteobacteria*. Before the intervention, the relative abundance of the above four major bacterial phyla was 37.69, 46.27, 7.37, and 4.74%, respectively; after the intervention, the relative abundance of the four major bacterial phyla was 46.97, 42.04, 4.60, and 3.36%, respectively (Figure 3A). After aerobic exercise intervention, the relative abundance of *Firmicutes* increased and the relative abundance of *Bacteroidetes, Actinobacteria*, and *Proteobacteria* decreased in people with Parkinson’s disease. The *Firmicutes*/*Bacteroidetes* (F/B) ratio was 0.8146 before the intervention, and the F/B ratio was 1.1172 after the intervention, which was elevated after the intervention.

At the class level, the most abundant bacterium was *Clostridia*, whose relative abundance was 25.62 and 36.60%, respectively, in the pre and post-training conditions, and the relative abundance of *Clostridia* increased (*p* = 0.043, Figure 3D).

At the family level, the relative abundance of *Lachnospiraceae*, *Ruminococcaceae*, and *Oscillospiraceae* was elevated after the intervention; and the relative abundance of *Prevotellaceae* and *Bacteroidaceace* was reduced (Figure 3B).

At the genus level, the relative abundance of *Faecalibacterium* and *Blautia* was increased after the intervention, and the relative abundance of *Prevotella_9* and *Bacteroides* was decreased (Figure 3C). The relative abundance of *Rosebuira* increased (*p* = 0.015, Figure 3E). At the phylum, order, family, and species levels, the changes in the relative abundance of gut microbiota after 8 weeks of aerobic exercise intervention were not significant.

### Aerobic exercise improves gut microbiota diversity in people with Parkinson’s disease

**Figure 4:**
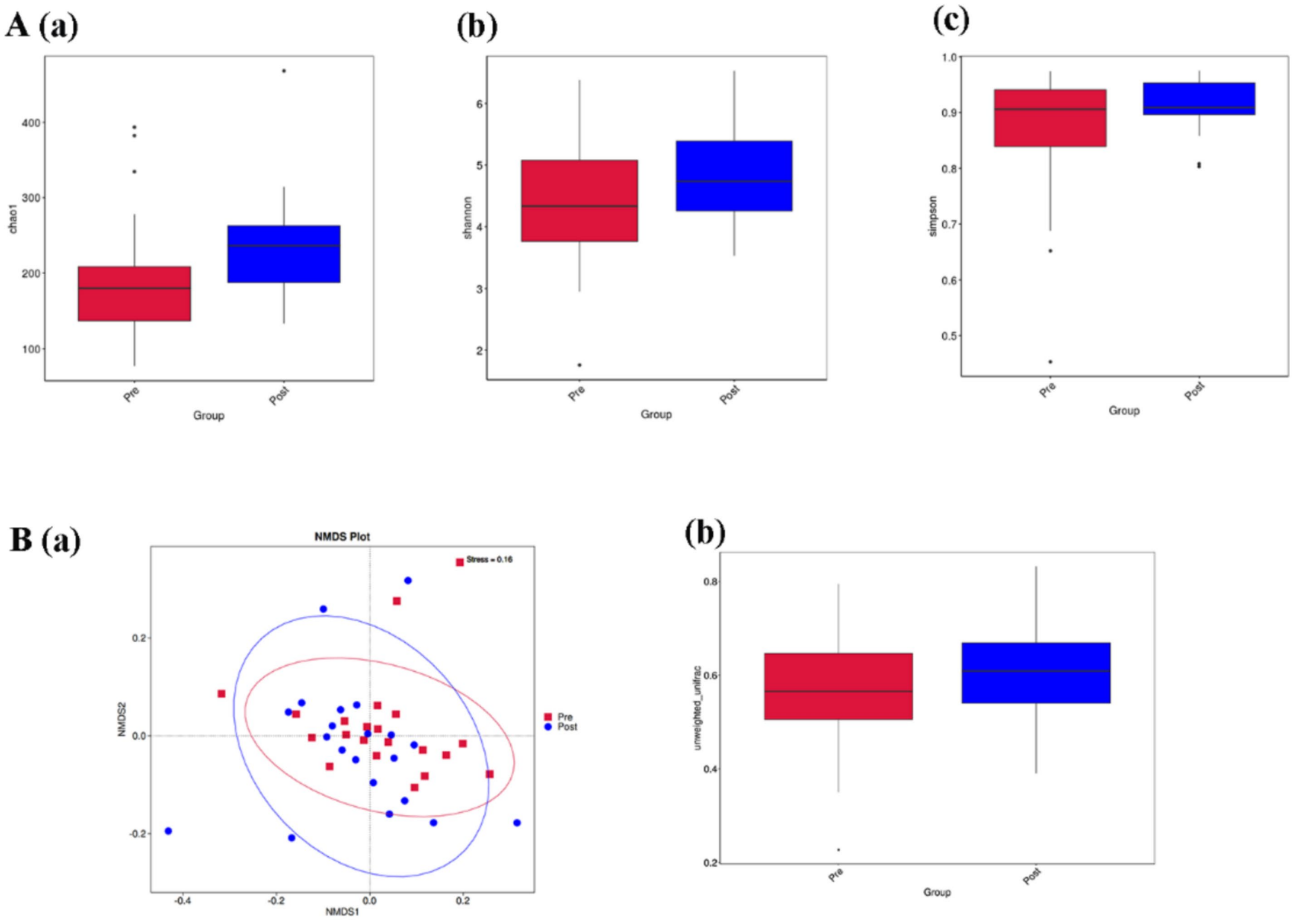
**(A,B)** Represent the comparison of alpha diversity and beta diversity before and after aerobic exercise intervention, respectively. **(A) (a)** Chao1 index; **(b)** Shannon index; **(c)** Simpson index; **(B) (a)** Non-Metric Multi-Dimensional Scaling (NMDS) plot based on Unweighted-unifrac distance; **(b)** Box plot of beta diversity comparison between groups based on Unweighted-unifrac distance box plot of beta diversity comparison between groups.

Alpha diversity is used to analyze the diversity of microbial communities within a sample. chao1 index, Shannon index, and Simpson index are some common indices of alpha diversity. chao1 index is used to estimate the total number of species contained in the community samples, which is a good response to the presence of low abundance species in the community; The Shannon index and Simpson index are both indicators used to describe the diversity (species richness and evenness) of the community. The Shannon index and Simpson index are both used to describe the community diversity, the higher the community diversity, the more even the species distribution, the larger the Shannon index and Simpson index. Alpha diversity index analysis showed that the Chao1 index of people with Parkinson’s disease was increased [*p* < 0.05, Figure 4A(a)], while the Shannon index and Simpson index of people with Parkinson’s disease were not statistically significant but also tended to increase after intervention [*p* = 0.09, *p* = 0.21, Figures 4A(b,c)].

Beta diversity is a comparative analysis of the microbial community composition of different samples. Figure 4B(a) is a Non-Metric Multi-Dimensional Scaling (NMDS) plot based on Unweighted-unifrac distance. A stress value less than 0.2 indicates that the NMDS accurately reflects the degree of variation between samples. The stress value for this study is 0.16, indicating that the graph can reflect the degree of difference between the samples before and after the intervention. Figure 4B(b) is a box plot of Beta diversity comparison between groups based on Unweighted-unifrac distance, and the results show that the Beta diversity index of people with Parkinson’s disease increased. (*p* < 0.05).

### Effect of aerobic exercise on bile acid metabolism

**Figure 5:**
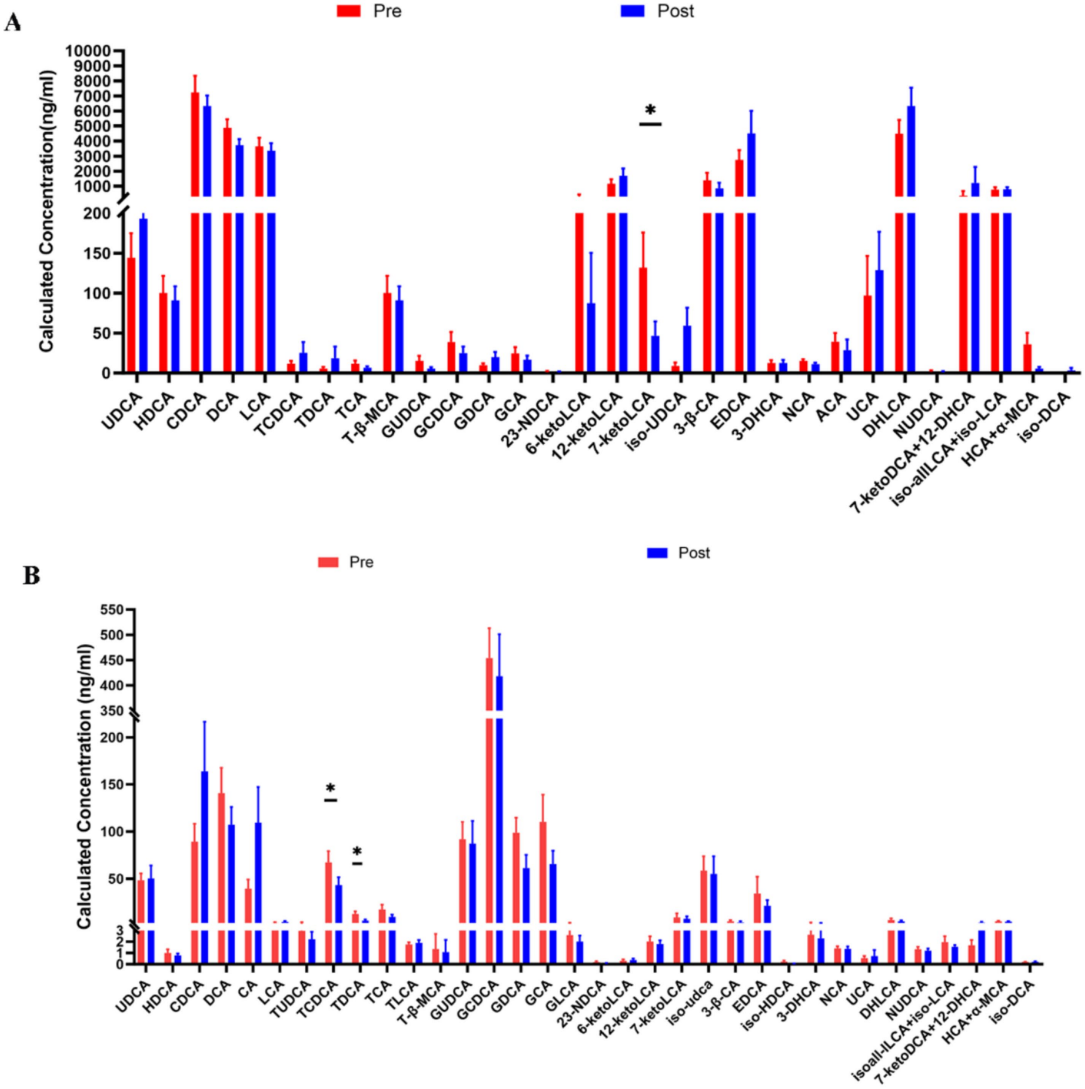
Comparison of fecal **(A)** and Serum bile acid **(B)** Metabolites in people with Parkinson’s disease before and after aerobic exercise intervention, *P < 0.05.

To determine the effect of aerobic exercise on bile acids in people with Parkinson’s disease, we examined fecal and serum bile acid metabolite levels in people with Parkinson’s disease using an LC–MS/MS system. Compared with pre-intervention, 7-ketoLCA concentrations were significantly decreased (Figure 5A) in people with Parkinson’s disease after the intervention; serum TCDCA and TDCA concentrations were significantly decreased (Figure 5B).

### The effect of aerobic exercise on inflammatory cytokine

**Figure 6:**
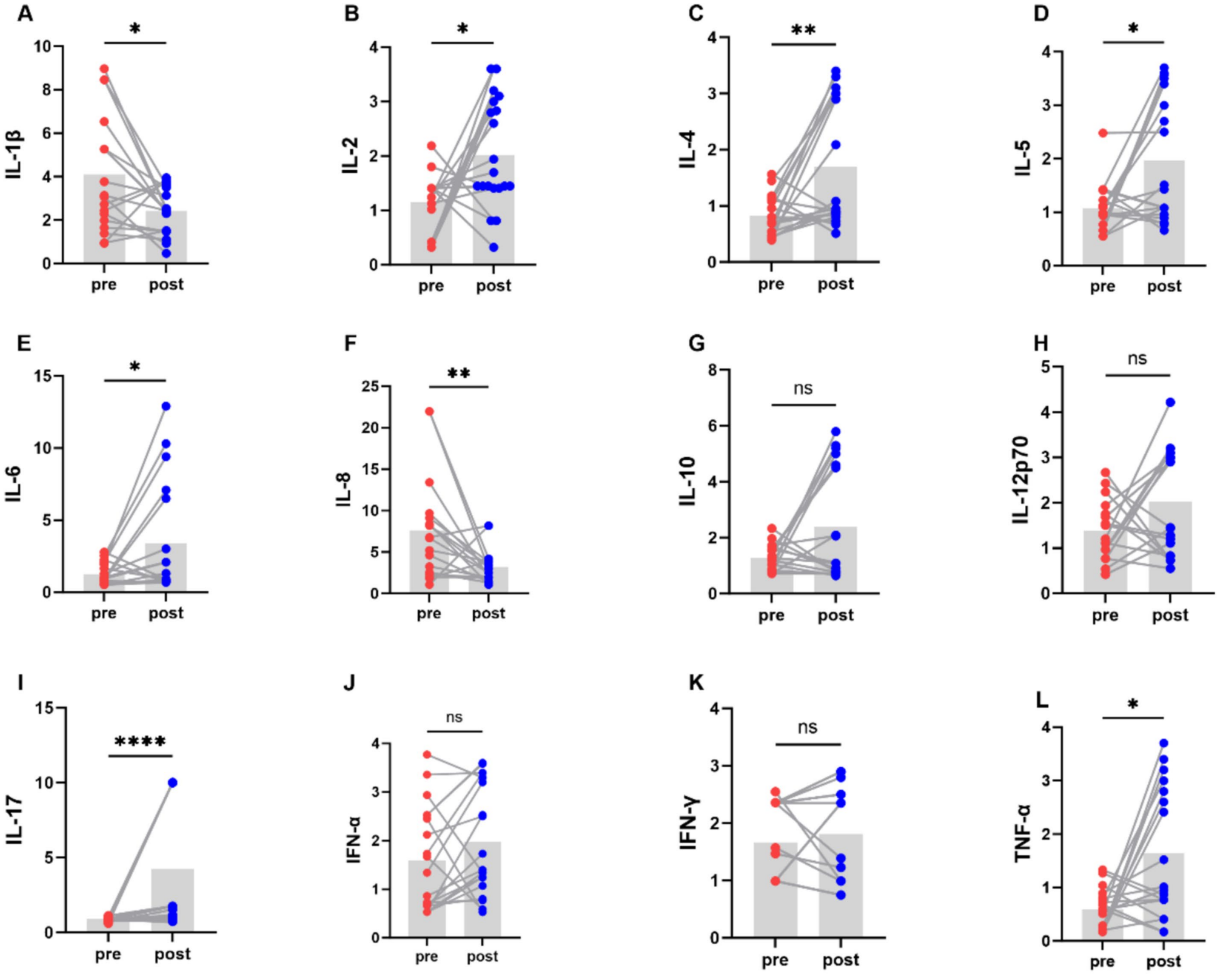
Comparison of levels of various cytokines in PD patients before and after aerobic exercise intervention, *P < 0.05; **P < 0.01; ****P < 0.0001.

We examined the levels of inflammatory factors in people with Parkinson’s disease using a 12-item T-cell factor test (Figure 6). The levels of the pro-inflammatory cytokines interleukin-1β and interleukin-8 decreased, while the level of the anti-inflammatory cytokine interleukin-4 increased.

### Correlation analysis

**Figure 7:**
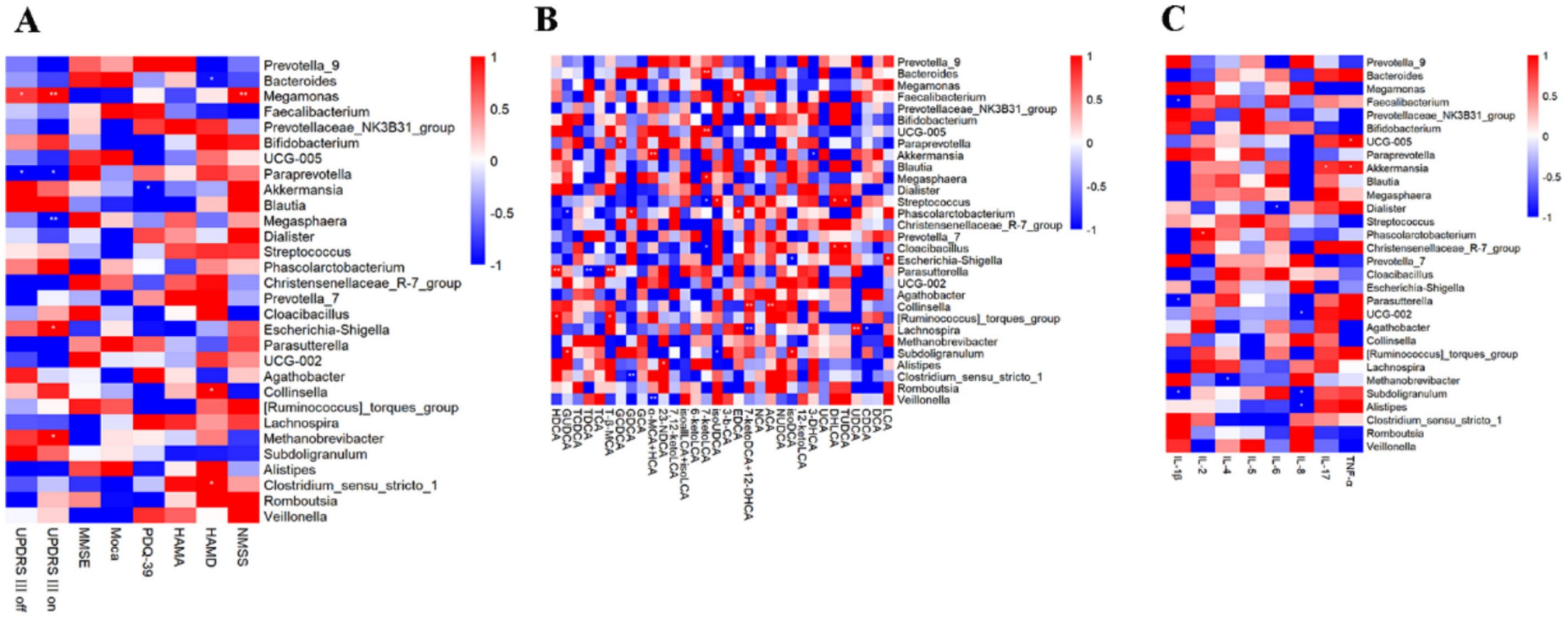
Correlation analysis. **(A)** Correlation analysis between the relative abundance of the top 30 genera in terms of abundance and changes in the PD scales. **(B)** Correlation analysis between the relative abundance of the top 30 genera in terms of abundance and changes in fecal bile acid levels. **(C)** Correlation analysis between the relative abundance of the top 30 genera in terms of abundance and changes in cytokines levels.

The correlation between the degree of change in the relative abundance of the top 30 genera in abundance and the degree of change in motor symptom scale scores in people with Parkinson’s disease before and after aerobic exercise interventions was analyzed by Spearman statistics (Figure 7A). The results showed that MDS-UPDRS III off-period scores were positively correlated with changes in the relative abundance of *Megamonas* but negatively correlated with *Paraprevotella*; MDS-UPDRS III on-period scores were positively correlated with changes in the relative abundance of *Megamonas*, *Escherichia. Shigella* and *Methanobrevibacter*, but negatively correlated with *Paraprevotella* and *Megasphaera*; Changes in cognitive scale scores exhibited no correlation with alterations in the relative abundance of gut microbiota. Conversely, changes in PDQ-39 scores demonstrated a negative correlation with *Akkermansia*. HAMD scores showed a negative correlation with *Bacteroides* while positively correlating with *Collinsella* and *Clostridium_sensu_stricto_1*. Additionally, NMSS scores were positively correlated with *Megamonas.*

To investigate the association between bile acids and intestinal flora, we analyzed the correlation between changes in the relative abundance of the top 30 genera and variations in fecal bile acid levels before and after aerobic exercise interventions in patients with Parkinson’s disease using Spearman’s correlation statistics (Figure 7B). The results indicated that 7-ketoLCA levels were positively correlated with *Bacteroides* and *Megasphaera* while showing a negative correlation with *Streptococcus* and *Cloacibacillus*.

To investigate the connection between gut microbiota and immune inflammation, Spearman statistical analysis was employed to examine the correlation between changes in the relative abundance of the top 30 bacterial genera before and after aerobic exercise intervention in people with Parkinson’s disease and the corresponding changes in inflammatory factor levels (Figure 7C). IL-4 showed a negative correlation with *Methanobrevibacter*. IL-6 presented a negative correlation with *Dialister*. IL-17 exhibited a positive correlation with *Akkermansia*. Finally, TNF-*α* showed a positive correlation with *UCG-005* and *Akkermansia*.

## Discussion

This study investigated the changes in clinical symptoms, gut microbiota diversity and composition, bile acid and inflammatory factor levels in patients with Parkinson’s disease following short-term aerobic exercise; it also explored the correlations between the relative abundance of gut microbiota and clinical scale scores, fecal bile acid levels, and changes in inflammatory factor levels. There are several findings in this study: first, aerobic exercise intervention improved motor and non-motor symptoms in people with Parkinson’s disease, which was manifested in the decrease of MDS-UPDRS-I, MDS-UPDRS-II, MDS-UPDRS-III, HAMA, HAMD, and NMSS scales scores; and the increase of MMSE, Moca scores. Second, aerobic exercise intervention modulated the relative abundance and diversity of gut microbiota in people with Parkinson’s disease, which was manifested as a decrease in the relative abundance of *Bacteroidetes* and an increase in the relative abundance of bacteria such as *Firmicutes*, *Clostridia*, and *Rosebuira* after the exercise intervention; and an increase in the Alpha diversity index (Chao1 index) and Beta diversity index. Third, aerobic exercise intervention can regulate the bile acid levels, specifically manifested by a significant decrease in the concentration of fecal 7-ketoLCA and a significant reduction in the concentrations of serum TCDCA and TDCA. Fourth, the levels of the pro-inflammatory cytokines interleukin-1β and interleukin-8 decreased, while the level of the anti-inflammatory cytokine interleukin-4 increased after aerobic exercise.

In this study, we found that the MDS-UPDRS-II and MDS-UPDRS-III scores of people with Parkinson’s disease tended to decrease after aerobic exercise intervention, indicating that aerobic exercise significantly improved the motor symptoms of PD. This is similar to the results of a previous study (9). Similarly, we also found that non-motor symptoms in people with Parkinson’s disease improved. Specifically, NMSS scores, Moca scores, HAMA scores, and HAMD scores improved after aerobic exercise interventions, which is similar to the results of Tollár’s study (32), indicating that cognitive functioning, level of anxiety and depression in people with Parkinson’s disease significantly improved after aerobic exercise interventions. Cognitive dysfunction can adversely affect the quality of life of people with Parkinson’s disease or even exacerbate their motor symptoms (33). Uygur’s study found that a six-week exercise intervention was effective in improving physical and cognitive function in patients with mild to moderate Parkinson’s disease (34). Although the subjects’ FOG-Q, Berg, PSQI, and Cleveland scale scores were not found to be statistically significant, there was a trend of improvement, which suggests that aerobic exercise has some improvement in symptoms such as freezing gait, balance function, sleep disturbances, and constipation in people with Parkinson’s disease and that perhaps longer intervention cycles or larger sample sizes are needed to obtain greater statistical. In conclusion, our study suggests that aerobic pedaling exercise is a relatively safe and feasible intervention for people with Parkinson’s disease, which may improve not only motor symptoms but also non-motor symptoms in terms of cognitive dysfunction and depression.

Our findings suggest that aerobic exercise improves motor and non-motor symptoms in people with Parkinson’s disease, but the mechanism of improvement warrants further investigation. Most studies have found differences in the gut microbiota between people with Parkinson’s disease and healthy controls, including upregulation of the abundance of some beneficial bacteria and decreased abundance of some pathogenic bacteria. For example, Li et al. (26) who summarized a large body of literature found that 53 family-level microorganisms and 98 genus-level microorganisms differed between people with Parkinson’s disease and healthy populations in 26 studies. This was characterized by an increase in *Enterococcaceae, Enterococcus, Lactobacillaceae, Lactobacillus*, *Verrucomicrobiaceae*, and *Akkermansia* and a decrease in *Lachnospiraceae*, *Prevotellaceae*, *Faecalibacterium*, and *Roseburia*. The intestinal flora is affected by a variety of factors, including environment, diet, and exercise; therefore, intestinal flora may be a potential target for intervention. Currently, there are fewer studies on the effects of aerobic exercise on the intestinal flora of people with Parkinson’s disease, and most of them are basic studies. Studies by Fan (19) and Wang (20), among others, have shown that aerobic exercise can regulate the composition of intestinal flora in PD mice. Therefore, we investigated the effect of aerobic exercise on the intestinal flora of people with Parkinson’s disease.

Our findings suggest that aerobic exercise modulates the relative abundance of gut microbiota in people with Parkinson’s disease. At the phylum level, the relative abundance of *Firmicutes*, increased, the relative abundance of *Bacteroidetes*, *Actinobacteria*, and *Proteobacteria* decreased, and the *Firmicutes*/*Bacteroidetes* ratio increased in people with Parkinson’s disease after the intervention, similar to the results of other studies (35). *Firmicutes* is known to contain many beneficial bacteria and is an important producer of butyrate (a type of short-chain fatty acid). Short-chain fatty acids play a positive role in maintaining intestinal barrier permeability, anti-inflammation, and host energy regulation (36). However, Morita (37) et al. found that aerobic exercise increased the relative abundance of *Bacteroides* in the intestines of their subjects. *Bacteroides* is an opportunistic pathogen and its effect on host health depends on the environment within the host’s gut. It prefers to survive in the colon under mildly acidic conditions (pH 6.7) and grows poorly at pH 5.5 (38). At the class level, the relative abundance of *Clostridia* was increased in people with Parkinson’s disease after the intervention. It is composed of Gram-positive bacteria in t *Firmicutes*, which make up a relatively large portion of the intestinal flora, accounting for 10–40% of the total number of bacteria in the intestinal microflora (39). *Clostridia* can utilize large quantities of a wide range of nutrients that cannot be digested by the host and produce metabolites such as short-chain fatty acids, secondary bile acids, and other metabolites, which in turn provide energy to the intestinal epithelium, maintain the stability of the intestinal barrier, and interact with the immune system (40). At the genus level, the relative abundance of *Rosebuira* was increased in people with Parkinson’s disease after the intervention. It consists of Gram-positive anaerobic bacteria belonging to the *Firmicutes* and the class *Clostridia*, which includes five well-characterized species, *Roseburia intestinalis*, *R. hominis*, *R. inulinivorans*, *R. faecis*, and *R. cecicola*, which are all capable of producing short-chain fatty acids (especially butyrate), which have positive effects on the organism (41). In addition, the relative abundance of groups such as *Lachnospiraceae*, *Ruminococcaceae*, *Oscillospiraceae*, and *Blautia* increased after the intervention; and the relative abundance of groups such as *Bacteroidaceae*, *Bacteroides*, *Prevotellaceae*, and *Prevotella* decreased. Similar to the findings of Erlandson et al., (42) there was an increase in *Bifidobacteriaceae*, *Oscillospiraceae*, *Anaerobacteria* spp. and a decrease in *Prevotella* in sedentary older adults after 24 weeks of exercise.

Higher Alpha diversity of gut microbiota was reported in male athletes compared to healthy male controls, which was the first report of exercise increasing gut microbial diversity in humans (43). Subsequent studies further demonstrated significant differences in gut flora between rugby players and sedentary healthy control populations at both functional and genetic levels (44). A basic study found that aerobic exercise had no significant effect on microbial Alpha diversity indices (Chao1 index and Shannon index) and Beta diversity (based on binary-jaccard distance) in the feces of PD mice (19). The results of our study showed that aerobic exercise intervention increased the Alpha diversity index (Chao1 index) and Beta diversity (based on unweighted-unifrac distance) in people with Parkinson’s disease. It has been found that increased colonic transport time leads to a decrease in biodiversity in the gut (45), which may be the key to the modulation of gut flora composition and diversity by aerobic exercise. Boytar et al. (46) investigated the effects of these variables such as frequency, intensity, duration, and type of exercise on changes in the gut microbiota in both healthy individuals and clinical populations, and the results of their study showed that ≥3 times per week (or weekly 150–270 min) of moderate- to high-intensity exercise for 30–90 min for ≥8 weeks may induce changes in the gut microbiota. Thus, longer intervention times or more intense interventions may produce more pronounced changes in gut microbiota.

To explore the relationship between gut microbiota and the improvement of clinical symptoms in people with Parkinson’s disease through aerobic exercise, we conducted a correlation analysis between the changes in the relative abundance of the top 30 genera before and after aerobic exercise intervention and the changes in scores of scales such as MDS-UPDRS-III, cognition, anxiety, and depression. The change in MDS-UPDRS-III scores was positively correlated with the change in the relative abundance of *Megamonas* and negatively correlated with *Paraprevotella*. The NMSS score was positively correlated with *Megamonas*. Among these, *Megamonas* belongs to the phylum Firmicutes and can ferment various carbohydrates, with its abundance increased in obese populations (47). The change in PDQ-39 scores after aerobic exercise intervention was negatively correlated with *Akkermansia*. It plays an important role in Parkinson’s disease, with functions such as regulating the intestinal barrier and modulating host immune responses (48), potentially indirectly improving the quality of daily life in People with Parkinson’s disease. Additionally, the HAMD score was negatively correlated with Bacteroides and positively correlated with *Collinsella* and *Clostridium_sensu_stricto_1*. Therefore, gut microbiota plays a significant role in the improvement of both motor and non-motor symptoms of Parkinson’s disease through aerobic exercise.

Bile acids also play a crucial role in the link between gut microbiota and Parkinson’s disease. For instance, Li et al. (30) found that gut dysbiosis is associated with elevated bile acids in Parkinson’s disease, with increased levels of secondary bile acids LCA and DCA in the ileum of people with Parkinson’s disease. LCA and DCA are highly hydrophobic, and their increase can induce pro-inflammatory and direct cytotoxic effects. Furthermore, bile acid levels are influenced by various factors such as diet, hepatobiliary diseases, exercise, gut microbiota, physiological state, and genetics. Our findings show that after aerobic exercise intervention, the levels of 7-keto lithocholic acid in the feces of people with Parkinson’s disease decreased, as did the serum levels of TCDCA and TDCA. In the bile acid metabolism process, LCA can undergo a series of oxidation reactions in the body to produce 7-ketoLCA. DCA can combine with taurine under certain conditions to form TDCA. Additionally, our results indicate that although most changes in bile acids did not reach statistical significance, there is a correlation between changes in gut microbiota and bile acid levels following aerobic exercise. Future research may need to strictly control diet, increase exercise intensity, or extend exercise duration to observe more significant results.

The effects of aerobic exercise interventions on the levels of inflammatory cytokine in people with Parkinson’s disease may be contradictory. The results of Jang et al. (49) showed that 8 weeks of endurance exercise inhibited pro-inflammatory cytokines (TNF-*α* and IL-1β) by down-regulation of α-Syn and attenuation of TRL2 signaling pathway which in turn improved the locomotor function in PD mice. The study of Moon et al. (50) demonstrated that a 6-week qigong intervention could reduce serum TNF-α levels in people with Parkinson’s disease. Soke et al. (51) found that 8 weeks of task-oriented training combined with aerobic exercise failed to alter serum TNF-α and IL-1β in people with Parkinson’s disease, which may be related to the older average age of the subjects, as the magnitude of exercise-induced cytokine changes decreased with age. Our research findings indicate that following exercise intervention, the levels of the pro-inflammatory cytokines interleukin-1β and interleukin-8 decreased, while the level of the anti-inflammatory cytokine interleukin-4 increased. In addition, levels of some pro-inflammatory cytokines IL-2, IL-5, IL-6, IL-17, and TNF-α were elevated. A possible reason for this could be that the concentration of pro-inflammatory cytokines may increase with age and disease progression. Secondly, acute exercise might affect autoimmune conditions, for example, an increase in lymphocytes (B cells and T cells) occurs during exercise or in the early recovery phase post-exercise, and is positively correlated with the intensity and duration of continued exercise (52).

Gut microbial dysbiosis is associated with an abnormal immune response, which is usually accompanied by an abnormal production of inflammatory factors (24). Dysbiosis of the intestinal flora leads to elevated levels of LPS in the gut, which in turn leads to intestinal inflammation, and LPS and inflammatory factors enter the bloodstream through the intestinal barrier, which in turn promotes CNS pathology in PD by the peripheral immune system and microglial cells of the brain (25). A study by Lin et al. (28) showed that there is a correlation between alterations in gut microbiology and alterations in plasma cytokine concentrations in people with Parkinson’s disease, with a positive correlation between *Bacteroides* was positively correlated with plasma TNF-*α*, and *Verrucomicrobiaceae* was positively correlated with plasma IFN-*γ* concentration. Mokhtarzade et al. (53) investigated the relationship between exercise-induced changes in the intestinal flora and changes in cytokines, and the results showed that there was a negative correlation between the counts of *Prevotella*, *Akkermansia Muciniphila*, and *Bacteroides* with the changes in IL-10 in patients with multiple sclerosis after a 6-month exercise intervention. In this study, *Akkermansia* is positively correlated with IL-17 and TNF-α. The potential mechanisms by which aerobic exercise improves clinical symptoms in people with Parkinson’s disease may be mediated through gut microbial and inflammatory factor pathways. However, the mechanisms underlying the interactions between aerobic exercise, gut flora, and Parkinson’s disease remain to be elucidated.

This study has several limitations. First, the lack of a control group does not allow the influence of spontaneous disease progression, placebo effect, or other confounding factors on the outcome to be completely ruled out. We propose a future large-scale, randomized controlled trial design with a control group that does not exercise or performs other types of exercise to confirm the specific efficacy of aerobic exercise. Second, the duration of the exercise intervention may be a significant factor affecting the outcomes, as we only conducted an eight-week exercise intervention. Future research may require higher exercise intensity and longer intervention periods to observe significant changes in gut microbiota, bile acids, and inflammatory cytokine levels. Third, after the completion of the eight-week exercise intervention, we did not have the participants cease exercising and conduct follow-up assessments, so it is unclear how long the improvements in these indicators can be maintained. Third, inadequate sample size, a larger sample size may be necessary to achieve greater statistical power, particularly for indicators showing a trend of change.

## Conclusion

Aerobic exercise is an effective therapeutic approach to improve clinical symptoms, increase the relative abundance of beneficial bacteria (such as *Clostridia* and *Rosebuira*) and Alpha-diversity index (such as Chao1 index) and Beta- diversity of intestinal flora, and modulate the levels of inflammatory factors in PD patients. Our study provides another potential mechanism by which aerobic exercise improves clinical symptoms in PD patients.

## Data Availability

The raw data supporting the conclusions of this article will be made available by the authors, without undue reservation.
